# Binding of CML-Modified as Well as Heat-Glycated β-lactoglobulin to Receptors for AGEs Is Determined by Charge and Hydrophobicity

**DOI:** 10.3390/ijms21124567

**Published:** 2020-06-26

**Authors:** Hannah E. Zenker, Malgorzata Teodorowicz, Arifa Ewaz, R.J. Joost van Neerven, Huub F.J. Savelkoul, Nicolette W. De Jong, Harry J. Wichers, Kasper A. Hettinga

**Affiliations:** 1 Food Quality & Design Group, Wageningen University & Research Centre, 6708 WG Wageningen, The Netherlands; hannah.zenker@wur.nl; 2 Cell Biology & Immunology, Wageningen University & Research Centre, 6700 AH Wageningen, The Netherlands; gosia.teodorowicz@wur.nl (M.T.); arifa.ewaz@wur.nl (A.E.); joost.vanneerven@frieslandcampina.com (R.J.J.v.N.); huub.savelkoul@wur.nl (H.F.J.S.); 3 FrieslandCampina, 3818 LA Amersfoort, The Netherlands; 4 Internal Medicine, Department Allergology & Clinical Immunology and Erasmus University Medical Centre Rotterdam, 3000 CA Rotterdam, The Netherlands; n.w.dejong@erasmusmc.nl; 5 Wageningen Food & Biobased Research, Wageningen University & Research Centre, 6708 WG Wageningen, The Netherlands; harry.wichers@wur.nl

**Keywords:** CD36, charge, Galectin-3, glycation, sRAGE, Nε-carboxymethyl lysine, milk protein

## Abstract

Intake of dietary advanced glycation end products (AGEs) is associated with inflammation-related health problems. Nε-carboxymethyl lysine (CML) is one of the best characterised AGEs in processed food. AGEs have been described as ligands for receptors present on antigen presenting cells. However, changes in protein secondary and tertiary structure also induce binding to AGE receptors. We aimed to discriminate the role of different protein modifications in binding to AGE receptors. Therefore, β-lactoglobulin was chemically modified with glyoxylic acid to produce CML and compared to β-lactoglobulin glycated with lactose. Secondary structure was monitored with circular dichroism, while hydrophobicity and formation of β-sheet structures was measured with ANS-assay and ThT-assay, respectively. Aggregation was monitored using native-PAGE. Binding to sRAGE, CD36, and galectin-3 was measured using inhibition ELISA. Even though no changes in secondary structure were observed in all tested samples, binding to AGE receptors increased with CML concentration of CML-modified β-lactoglobulin. The negative charge of CML was a crucial determinant for the binding of protein bound CML, while binding of glycated BLG was determined by increasing hydrophobicity. This shows that sRAGE, galectin-3, and CD36 bind to protein bound CML and points out the role of negatively charged AGEs in binding to AGE receptors.

## 1. Introduction

β-lactoglobulin (BLG) is the main component of the whey protein fraction that contributes ~10% to the total protein in cow’s milk and has been described as one of the major allergens in cow’s milk [1]. Moreover, it is one of the few proteins in cow’s milk that is not present in human breastmilk. Its amino acid sequence consist of 162 amino acids, including 16 lysine residues, 3 arginine residues, and 1 free cysteine [2], which makes it prone to glycation via the Maillard reaction (MR) as well as heat induced unfolding. This may result in the formation of advanced glycation end products (AGEs) and irreversible structural changes of BLG upon heating. Formation of AGEs as a result of the MR can occur endogenously or exogenously. Exogenous AGEs have a source in thermally processed foods, where protein is heated in the presence of reducing sugars. This is considered to contribute to 10% of the AGE pool in the body [3]. There is a growing body of evidence that AGEs contribute to a number of inflammation-related health issues such as chronic inflammation, atherosclerosis, and allergy [4,5,6]. In fact, in vivo animal studies with glycated food proteins confirmed their potential to activate inflammatory pathways and showed the contribution of high dietary intake of AGEs on inflammation-mediated health implications [7,8]. Furthermore, it was reported that glycation of food proteins enhances their uptake by antigen presenting cells (APCs) and affects T-cell proliferation [9,10,11].

Antigen uptake and initiation of immunological responses to AGEs is mediated by AGE-receptor/ligand interactions on the surface of APCs [12]. A number of AGE-binding receptors on the surface of innate immune cells have been described in previous studies, such as the receptor for AGEs (RAGE), CD36, galectin-3 as part of the AGE-R complex, scavenger class A type I and type II [13,14,15,16,17]. Next to AGEs, these pattern recognition receptors can also bind to other structures and some share other ligands such amyloid beta for RAGE and CD36, as well as bacterial lipopolysaccharides for RAGE and galectin-3. [18,19,20]. Lysine modification with Nε-carboxymethyl lysine (CML) is one of the most abundant as well as one of the best characterised AGEs in processed milk [21,22]. CML has been the target of previous studies, determining the effect of AGEs in receptor mediated immune responses [23,24,25]. Only a few studies looked at the role of specific AGEs while limiting 3D-folding modifications during the glycation process [23,26]. However, disentangling these effects is crucial, as protein aggregation, heating, and glycation induced increases of hydrophobicity and β-sheet structures have also been reported as determinants for uptake of glycated food proteins by macrophages and the binding to AGE receptors [27,28,29]. In this study, chemical modification of the milk allergen BLG was performed to obtain a specific modification of lysine to CML, which was then compared to BLG glycated with lactose below the denaturation temperature of BLG, to avoid heat-induced structural changes of the protein. Binding to the soluble form of RAGE (sRAGE), which shows identical binding to the extracellular ligands as RAGE [30], as well as binding to CD36 and galectin-3 was assessed with inhibition ELISA tests to assess the respective role of various protein modifications in receptor binding.

## 2. Results

### 2.1. Chemical Modification and Glycation

To analyse binding of CML on BLG to receptors, the protein was chemically modified with glyoxal to CML (BLG-CML). To distinguish the effect of lysine blockage via the MR with lactose, without heat-induced structural changes, BLG was glycated with lactose (BLG-Lac) at 60 °C, at a_w_ 0.23 for 12, 24, and 48 h. Moreover, subsequent chemical CML modification of glycated BLG was used to evaluate whether this changed receptor binding epitopes. The level of CML modification was determined using LC-MS/MS (Table 1).

**Figure 1:**
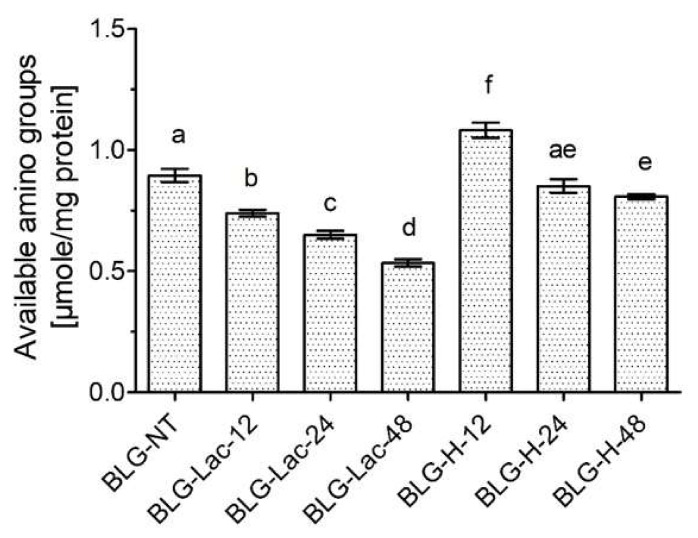
Available amino groups of β-lactoglobulin (BLG), non-treated (NT), glycated with lactose (Lac) by heating at 60 °C for 12, 24, and 48 h, as well as heated at 60 °C for 12, 24, and 48 h in the absence of any sugars (H). Error bars represent standard deviation of technical replicates (*n* = 3). Statistical differences were tested using one-way ANOVA with Tukey post-hoc test. Significant differences were considered at *p* < 0.05, where two variables have different letters if they are significantly different.

The level of CML-modified lysine and arginine residues increased with a higher ratio of glyoxylic acid/BLG, resulting in 17% modified residues in BLG-CML-1, 34% in BLG-CML-3, and 38% in BLG-CML-5. BLG heated in the absence of lactose (BLG-H) and BLG heated in the presence of lactose (BLG-Lac) contained much lower levels of CML than BLG-CML samples, regardless of the heating time. Modification of BLG with CML after glycation (BLG-Lac-CML) showed higher levels of CML modification with shorter heating time, resulting in 28%, 31%, and 16% CML-modified residues in BLG-Lac-12-CML, BLG-Lac-24-CML, and BLG-Lac-48-CML, respectively. To determine the extent of heat- and glycation-induced modification in BLG-H and BLG-Lac, free amino groups were quantified using the o-phthaldialdehyde (OPA) assay (Figure 1).

When BLG was heated in the presence of lactose, the number of free amino groups decreased with longer heating time. At the same time, heating in the absence of lactose first increased the levels of free amino groups after 12 h of heating (BLG-H-12), but subsequently decreased with prolonged heating time to the level of BLG-NT.

### 2.2. Structural Changes

**Figure 2:**
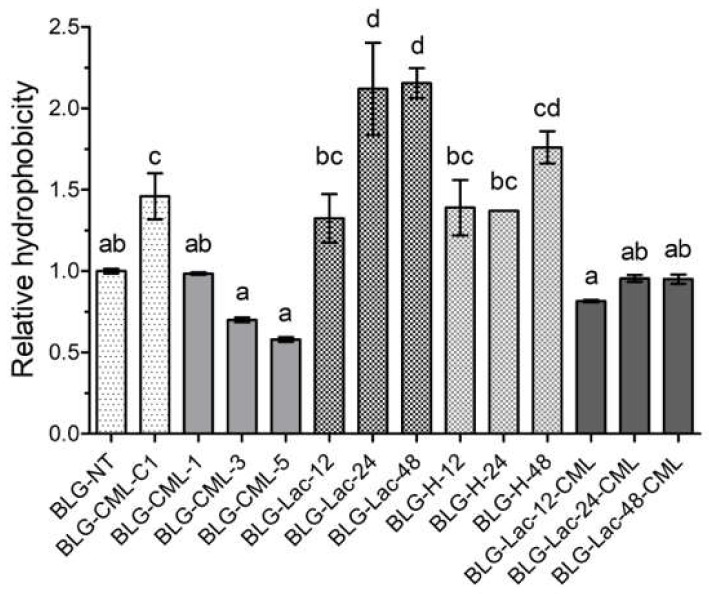
Hydrophobicity of β-lactoglobulin (BLG) samples relative to non-treated (NT) BLG, chemical modification control without addition of glyoxylic acid (C1), chemically modified to introduce Nε-carboxymethyl lysine (CML) at different degree of modification (1, 3, and 5), glycated with lactose (Lac) by heating at 60 °C for 12, 24, and 48 h; heated at 60 °C for 12, 24, and 48 h in the absence of any sugars (H), and glycated by heating with lactose followed by CML induction with glyoxal. Error bars represent standard deviation of technical replicates (*n* = 2). Statistical differences were tested using one-way ANOVA with Tukey post-hoc test. Significant differences were considered at *p* < 0.05, where two variables have different letters if they are significantly different.

Hydrophobicity relative to untreated BLG was measured using the 8-anilino-1-naphthalenesulfonic acid (ANS)-assay (Figure 2).

BLG-CML did not show significant differences in relative hydrophobicity compared to BLG-NT, independent of the level of modification. At the same time, BLG-Lac showed significantly higher levels of hydrophobicity compared to BLG-NT after 24 and 48 h heating time. Heating in the absence of lactose did not change hydrophobicity of BLG until 24 h of heating, however it increased after 48 h heating. Modification of BLG-Lac samples with glyoxylic acid to form additional CML, resulted in decreased hydrophobicity of BLG-Lac-24-CML and BLG-Lac-48-CML compared to the respective BLG-Lac samples and reached comparable levels as for BLG-NT.

Circular dichroism (CD) spectra were monitored to determine changes in the secondary structure of BLG by heating, glycation, and chemical modification, respectively (Figure S1). The CD-spectra of all samples showed no deviation from the spectra of BLG-NT.

**Figure 3:**
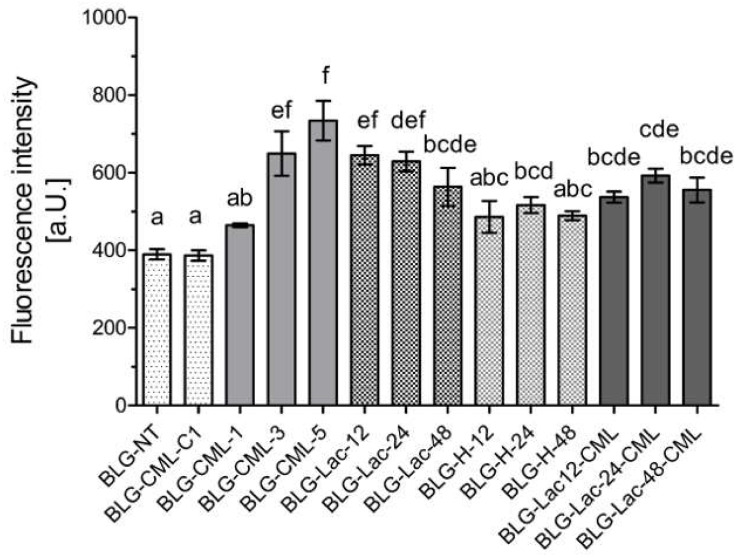
Thioflavin-T assay of β-lactoglobulin (BLG), non-treated (NT), control for chemical modification (C1), chemically modified BLG to introduce Nε-carboxymethyl lysine (CML) at different levels of CML, glycated with lactose (Lac) by heating at 60 °C for 12, 24, and 48 h; heated at 60 °C for 12, 24, and 48 h in the absence of any sugars (H), and glycated by heating with lactose followed by CML induction with glyoxal. Fluorescence intensity was corrected for the autofluorescence of the samples and fluorescence of the blank. Significant differences were tested using one-way ANOVA with Tukey post-hoc test, considered as significant at *p* < 0.05 between technical replicates (*n* = 2), where two variables have different letters if they are significantly different.

Thioflavin T (ThT) assay was performed to monitor changes in the level of β-sheets upon treatment of BLG (Figure 3). BLG-CML-3 and BLG-CML-5 revealed significantly higher fluorescence intensity compared to BLG-NT, with the tendency of higher fluorescence intensity with increasing level of modification in BLG-CML samples. Additionally, BLG-Lac samples showed a higher signal compared to BLG-NT. BLG-H-24 has significantly higher fluorescence intensity compared to BLG-NT but not compared to the other BLG-H samples. BLG-Lac-CML samples showed higher fluorescence intensity than BLG-NT, however fluorescence intensity did not differ between BLG-Lac-CML samples.

**Figure 4:**
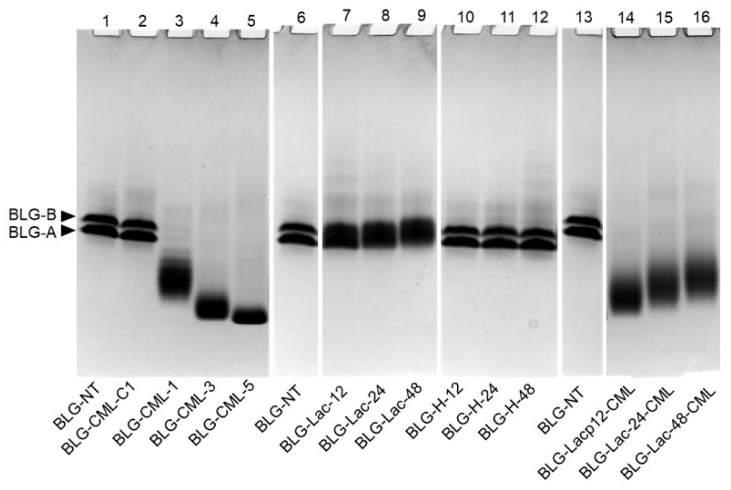
Native-PAGE of β-lactoglobulin (BLG), non-treated (NT), chemical modification control (C1), chemically modified to introduce Nε-carboxymethyl lysine (CML) at different degree of modification (1, 3, and 5), glycated with lactose (Lac) by heating at 60 °C for 12, 24, and 48 h; heated at 60 °C for 12, 24, and 48 h in the absence of any sugars (H), and glycated by heating with lactose followed by CML induction with glyoxal. BLG-NT of lane 1 and 13 are identical and were on the same gel as samples in lane 2–5 and lane 14–16, whereas BLG-NT in lane 6 was run on the same gel as samples in lane 7–12.

Gel-electrophoretic separation was conducted under native conditions to monitor the occurrence of protein aggregation after the treatments (Figure 4). This showed that no aggregation had occurred.

BLG-NT and BLG-C1 (lane 1 and lane 2) showed two distinct bands corresponding to the variant A and B that show slight differences of their isoelectric point and therefore can be separated via native gel electrophoresis [31]. BLG-CML samples (lane 3–5) showed one broad band and higher level of CML modification caused further migration through the gel accompanied with decreased broadening of the band. Whereas in SDS-PAGE under reducing conditions, only minimal differences in migration behaviour of BLG-CML samples were observed (data not shown). BLG-Lac (lane 7–9) samples showed one broad band at the same position as the two bands of BLG-NT. Only BLG-Lac-48 showed a slightly lesser migration. BLG-H (lane 10–12) samples showed no difference to BLG-NT, independent from the heating time. BLG-Lac-CML (lane 14–16) samples showed one broad band at a lower position compared to BLG-NT and BLG-Lac samples. As in BLG-CML samples, the BLG moved faster through the gel upon higher level of CML modification.

### 2.3. Receptor Binding

**Figure 5:**
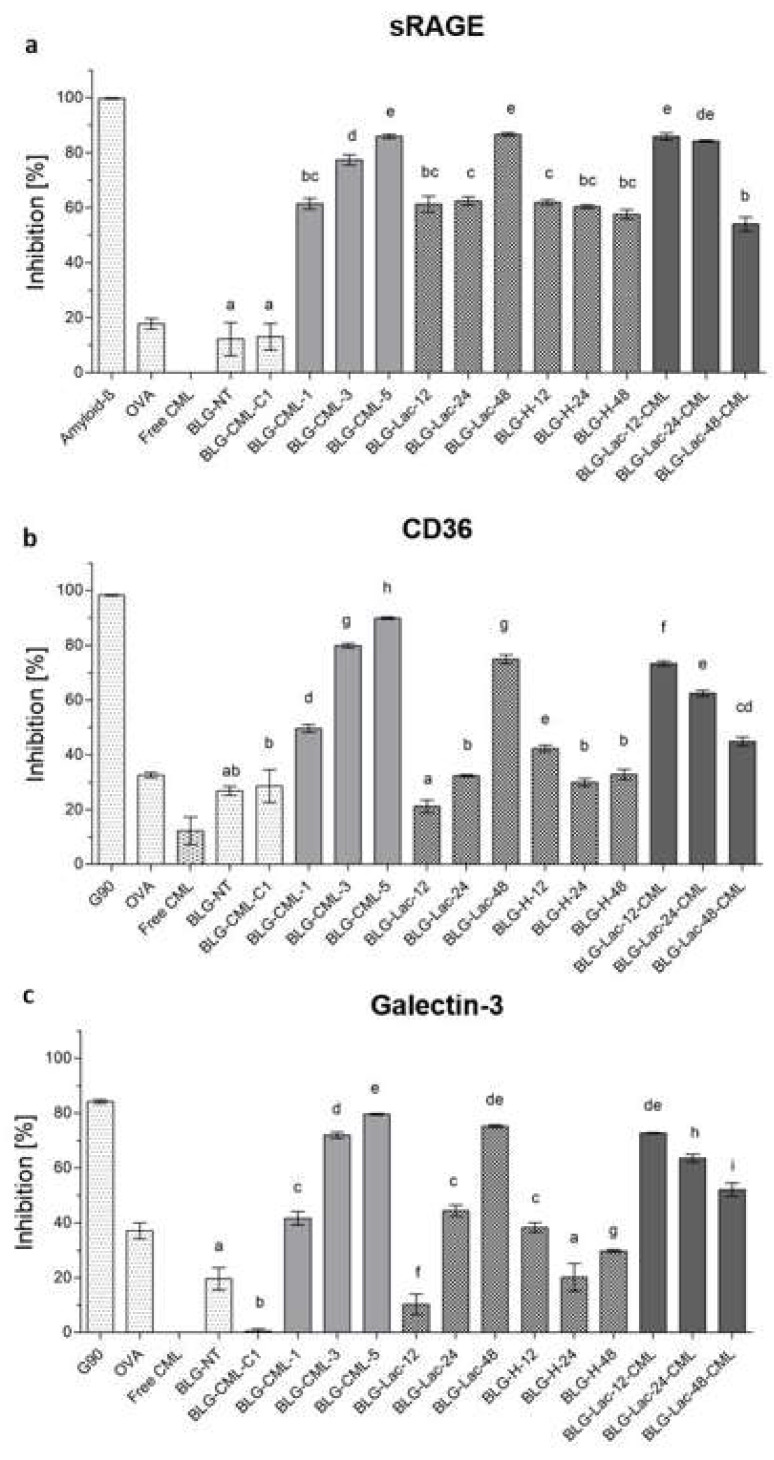
Inhibition ELISA for binding of (**a**) sRAGE, (**b**) CD36, and (**c**) Galectin-3 to β-lactoglobulin (BLG), non-treated (NT), control for chemical modification (CML-C1), chemically modified BLG with Nε-carboxymethyl lysine (CML) at different levels of CML, glycated with lactose (Lac) by heating at 60 °C for 12, 24, and 48 h; heated at 60 °C for 12, 24, and 48 h in the absence of any sugars (H), and glycated by heating with lactose followed by CML induction with glyoxal, with ovalbumin (OVA) as negative control, and glycated soy protein (G90) as positive control for CD36 and galectin-3, as well as amyloid-β as positive control for sRAGE. Protein concentrations were set to 100 µg/mL, while free CML was used at 2 mg/mL. Significant differences were tested using one-way ANOVA with Tukey post-hoc test, considered as significant at *p* < 0.05 between technical replicates (*n* = 3), where two variables have different letters if they are significantly different.

Binding of CD36, galectin-3, and sRAGE was determined with inhibition ELISA, where higher inhibition indicates more binding to receptor ligands (Figure 5).

All receptors showed increased binding to the BLG-CML samples compared to BLG-NT, which increased with higher CML levels. At the same time binding to free CML was only observed for CD36, however this was lower than the binding to OVA. BLG-Lac samples showed higher binding upon prolonged heating time, however only starting after 24 h for both CD36 and galectin-3. In contrast, sRAGE binding increased already for BLG-Lac-12, remained on the same level in BLG-Lac-24 and then increased again for BLG-Lac-48. BLG-H samples revealed no heating time-dependent trend for binding to all receptors and, overall, displayed either similar or lower binding compared to the BLG-Lac sample heated for the same duration. For CD36, only BLG-H-12 showed higher binding than BLG-NT, while for galectin-3 both BLG-H-12 and BLG-H-48 showed higher levels of binding. For sRAGE, the BLG-H samples all showed a level of binding that was similar to the BLG-Lac samples, and significantly increased compared to BLG-NT. BLG-Lac-CML samples showed higher binding the more CML was present in the sample, which followed a stepwise increase for binding to CD36 and galectin-3, while sRAGE showed similar binding to BLG-Lac-12 and BLG-Lac-24 and only decreased for BLG-Lac-48.

**Figure 6:**
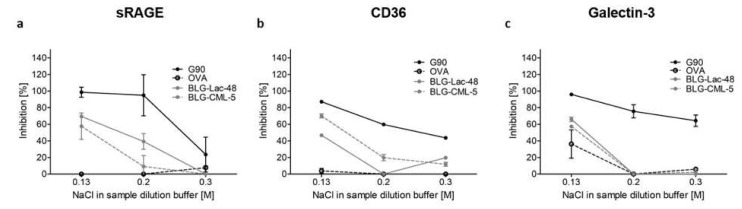
Inhibition ELISA for binding of (**a**) sRAGE, (**b**) CD36, and (**c**) galectin-3 to β-lactoglobulin (BLG), chemically modified BLG with Nε-carboxymethyl lysine (BLG-CML-5), BLG dry heated in the presence of lactose (Lac) for 48 h using different concentrations of sodium chloride (NaCl) in the sample dilution buffer. Protein concentrations were set to 100 µg/mL. Error bars represent standard deviation of technical replicates (*n* = 3).

Receptor binding with different concentrations of sodium chloride (NaCl) was performed to assess the role of charge in binding of BLG-CML-5 and BLG-Lac-48 to AGE receptors (Figure 6).

With increasing salt concentration of the sample dilution buffer to block the negative charge of CML, the binding to all receptors of BLG-Lac-48 and BLG-CML-5 decreased. For CD36 and galectin-3 no binding was observed already at 0.2 M NaCl in the sample dilution buffer, while sRAGE showed a gradual decrease in binding from 0.13 M to 0.3 M NaCl. Additionally, the positive control showed lower binding with increasing NaCl concentration, starting at 0.2 M NaCl for CD36 and galectin-3, while sRAGE showed a decrease only at 0.3 M NaCl.

## 3. Discussion

CML is not only the most commonly used marker of MR in food but also an important biomarker of protein modifications in vivo [32,33]. CML modifications in vivo were shown to contribute to increased levels of inflammation markers in serum [34,35]. However, disentangling the effect of the presence of AGEs and 3D-changes of the protein structure upon heating induced glycation was often not possible. Recent studies highlighted the importance of separating the effect of aggregation, heating-induced exposure of hydrophobic as well as β-sheet structures and the formation of AGEs in order to explore the role of AGEs in inflammation [27,28,29]. In this study, BLG was chemically modified with glyoxylic acid to induce CML and structural changes were closely monitored. The binding to sRAGE, CD36, and galectin-3 was evaluated to assess the immunoreactive potential of CML-modified proteins.

Modification of BLG with glyoxylic acid resulted in the formation of CML-modified BLG without changing the 3-D structure of the protein. This was illustrated by the increasing CML levels at higher ratio of glyoxylic acid to BLG (Figure 1), lack of additional structural modifications (Figure S1), and aggregation (Figure 4). Levels of CML in liquid and powdered dairy products have been reported in a wide range between 2–210 µg/g protein, where already small amounts are found in raw milk and increase with heating intensity and prolonged storage at higher temperatures (up to 50 °C for 4 months) [36]. In contrast, during the incubation of BLG in the presence of lactose only low amounts of CML were formed which was expected for the mild heat treatment and the short heating time (60 °C, for maximum 48 h). However, free amino groups decreased with prolonged incubation times (Figure 1), indicating that the MR occurred but did not proceed beyond the early stage, most likely resulting in the formation of other MR products (MRPs) than CML. The heating conditions used to induce protein glycation as well as the MR itself can promote protein aggregation [37]. This is a crucial determinant as it has been shown before that heating- and glycation-induced aggregation of BLG results in the formation of sRAGE binding ligands [28,29]. Native PAGE confirmed that in this well-defined system no aggregates were formed in any of the samples (Figure 4). However, faster movement through the native PAGE (Figure 4) also indicated that protein charge changed, especially in BLG-CML and BLG-Lac-CML. This was confirmed by the same mobility of all BLG-CML samples in SDS-PAGE gels where charge is not affecting mobility of the proteins (data not shown). The different charge seemed to be dependent on the CML content as CML gives an additional negative charge to each modified lysine. The broadening of the bands, being most distinct in BLG-CML-1 and the BLG-Lac samples, indicated the presence of a wide range of molecules modified differently, in extent and/or position.

Further structural analysis revealed that the chemical modification of BLG with CML did not result in CML level-dependent changes in hydrophobicity (Figure 2) and of the secondary structure (Figure S1). This is also in line with previous findings on the model protein ovalbumin which was chemically modified following the same procedure [23]. Additionally, BLG-Lac and BLG-H samples showed no changes in secondary structure compared to BLG-NT, which is in line with the results of Enomoto et al. [38] who reported that neither glycation nor heating under dry heating conditions at 50 °C significantly affected the secondary structure of BLG. However, the ThT-assay showed an apparent increase of β-sheet structures with increasing level of modification in BLG-CML samples, which is in contrast with the CD measurements (Figure S1). It is most commonly accepted that ThT binds to the cross β-structure of amyloid fibrils and thus is suitable to give an indication about their presence and relative quantities. However, it has also been shown to bind to hydrophobic structures [39]. This could explain the discrepancies between CD measurements (Figure S1) and ThT-assay (Figure 3) for BLG-Lac and BLG-H samples as they showed increased hydrophobicity (Figure 2). Moreover, ThT is positively charged and the signal intensity of ThT-fibril/protein complexes can be affected by the charge state of the ThT ligand [40]. Native PAGE (Figure 4) indicated that the chemically modified samples were more negatively charged the higher the level of CML. This is in line with the data of ThT binding assuming that in this case ThT interacts with the negative charge. This could possibly explain the discrepancies between the results from CD (Figure S1) and ThT measurements (Figure 3) for BLG-CML samples. This interpretation is supported by the general opinion that the chemical modification with glyoxylic acid is considered as a very specific method that solely results in CML modification of lysine residues without affecting the secondary structure [23]. BLG-Lac and BLG-H samples showed significant differences in surface hydrophobicity after 24 h incubation and 48 h incubation, respectively (Figure 2). This is in line with the findings of Morgan et al. [41] who showed that ANS binding increases upon dry heating of BLG (relative humidity of 65%, at 50 °C) and is even higher when BLG is heated in the presence of lactose. Structural changes of BLG-Lac samples were also confirmed by the native PAGE (Figure 4), that showed a broader band for BLG-Lac samples. However, this could also be the result of glycation-induced side-chain modifications on BLG-Lac samples, as indicated by the OPA-assay (Figure 1). To summarise, chemical modification of BLG did not result in detectable 3D-structural changes but did induce a negative charge to the lysine residues. At the same time, glycation of BLG increased the surface hydrophobicity (Figure 2). However, after chemical modification of BLG-Lac samples with CML, hydrophobicity decreased again, which is possibly related to the addition of a negative charge on lysine residues.

Dietary AGEs have previously been described as potent ligands for a number of cell surface receptors expressed on APCs including RAGE, CD36 and the extracellular unit of the AGE-R complex galectin-3 [42]. However, the knowledge on the particular binding sides of these receptors to MR-modified proteins is very limited because of complexity of the structural rearrangements during the heat treatment and glycation. For instance, heating that is required to form AGEs via the MR, as well as the MR itself, promote protein aggregation which was already shown to induce an interaction with sRAGE [28,29]. Structural analysis of BLG-CML showed that the tertiary and secondary structure of BLG is maintained upon modification. Moreover, no aggregates were observed on the native PAGE excluding protein unfolding or aggregation as the reason of increased binding to the receptors observed for BLG-CML. Both CML modified samples (BLG-CML and BLG-Lac-CML) showed a CML content-dependent increase of binding to all tested receptors (Figure 5), indicating the role of this specific AGE in promoting the binding to the selected receptors. This is in agreement with previous literature which described CML as ligand for sRAGE in vivo and in vitro [24,43]. However, glycation of the tested proteins was not selective for CML and structural changes were not monitored.

The investigated receptors are multiligand receptors that show binding to several structural elements. The role of amyloid structures, aggregation and hydrophobicity has previously been discussed in relation to binding to these receptors and cellular uptake in macrophages as well as a wide spectrum of lectin structures for galectin-3 [20,27,29,44,45]. Ohgami et al. [14] showed that CD36 in CHO-CD36 cells binds to AGE-OVA that carried a significant amount of CML, while for galectin-3 Vlassara et al. [17] reported that it shows high affinity binding to glycated bovine serum albumin (BSA) with unknown CML content. The results presented in this study are the first that directly show the role of CML in binding to CD36 and galectin-3. Based on the results of native PAGE and inhibition ELISA with various salt concentrations (Figure 6), it is hypothesized that the negative charge of CML is the crucial determinant to induce binding to sRAGE, CD36, and galectin-3. For sRAGE this is in agreement with the findings by Xue et al. [46], who showed that CML-modified peptides and CML-modified BSA bind to the positively charged cavity within the V domain of sRAGE. In contrast, a study conducted by Buetler et al. [26] showed that BLG which had 10–40% of its lysine chemically modified to introduce CML did not show binding of antibody-captured GST-RAGE by using the Biacore technique. However, to evaluate the binding, a buffer containing 5 mM calcium chloride was used in this study. Comparable to the effect of NaCl, where the positively charged sodium cation can interact with the negative charge of CML, this could decrease sRAGE binding (Figure 6). As reviewed by Collot-Teixeira et al. [45], CD36 binds to oxidised low density lipoprotein (oxLDL) which is suggested to occur via a positively charged moiety that binds to negatively charged ligands, such as oxLDL. According to these authors, the binding site within CD36 for oxLDL and AGEs are identical. Moreover, the role of charge in binding to murine CD36 ectodomain has also been postulated for diacylglycerol ligands [47], thus supporting the hypothesis that also for CD36 the negatively charged CML moiety of BLG-CML is essential for binding to the receptor. Galectins in general bind to carbohydrates, which is considered to occur as a result of hydrophilic interactions via hydrogen bonds, and hydrophobic interactions, specifically the CH-π interaction [48]. This explains the binding affinity of galectin-3 to lectins and lipopolysaccharides but not to BLG-CML with the hypothesis of a charge-dependent recognition. The binding mechanism of ligands to galectin-3 is in many cases unknown, emphasising the importance of further studies to better understand the binding affinity of galectin-3 and its interaction with protein-bound CML.

Interestingly, the binding to the AGEs receptors tested here also increased with longer incubation times of BLG-Lac samples, indicating that glycation with other MRPs than CML also plays a role in modulating the receptor recognition of BLG, while CML levels were much lower compared to BLG-CML samples (Table 1). This could be explained by the increasing hydrophobicity of BLG-Lac samples (Figure 2), which has been shown to promote the uptake of heated and glycated BLG in macrophages [27]. At the same time, BLG-Lac-CML samples showed no increase in hydrophobicity compared to BLG-NT (Figure 2) and lower levels of CML compared to BLG-CML samples (Table 1), while binding to the receptors was still high (Figure 5). This suggests that other MRPs, that induce a negative charge on lysine residues, could be involved in the increased binding to the AGE receptors. This is also supported by the decreasing binding of BLG-Lac-48 with increasing salt concentration and highlights the importance to closely monitor the degree of glycation in processed food.

In this study, it was shown that BLG bound CML is a ligand for the receptors present on APCs: RAGE, CD36, and galectin-3. Although the levels of CML induced in this study are higher than what is normally observed in processed milk and dairy products, this study suggests a role of CML in immunomodulation by dietary AGEs. Moreover, this study demonstrated that not only CML but also other MR-induced modifications result in binding to AGE receptors, because glycated BLG with low levels of CML also induced binding to these receptors. The role of dietary AGEs in inflammatory disease is still under debate due to lacking possibilities to differentiate between exogenous and endogenous AGEs, limited knowledge of the metabolic transit, and possible biases of some in vivo studies for instance by high caloric index of the study material as well as the presence of reactive oxidative species [49]. It is possible that smaller MR-modified digestive peptides reach the mucosal immune system in the gastrointestinal tract [50] and that intact BLG is sampled from the intestinal lumen via DCs and thus can be involved in mucosal immunity [51]. However, further studies are needed to evaluate the bioavailability of CML modified protein as well as CML modified digestive peptides and their possible immunomodulatory effect in vivo.

## 4. Materials and Methods

### 4.1. Chemicals

Lyophilised BLG from bovine milk (>90% purity) was purchased from Merck (Darmstadt, Germany). Novex™ Wedge Well™ 14% tris-glycine gel, Novex™ tris-glycine native sample buffer (2x), and Novex™ tris-glycine native running buffer (10x) were purchased from Thermo Fischer (Waltham, MA, USA). Coomassie brilliant blue R-250 was obtained from Biorad (Hercules, CA, USA). CML and CML-d2 were purchased from Polypeptide laboratories (Strasbourg, France). MS grade acetonitrile was purchased from Actu-All chemicals (Oss, The Netherlands). Ultra-pure water was prepared by an ELGA LabWater Purelab Plus water system (Celle, Germany). Soluble Advanced Glycation End Product-Specific Receptor Human E. coli (RD172116100) was purchased from Biovendor (Brno, Czech Republic). Anti-RAGE antibody (monoclonal mouse IgG2B clone, MAB11451), recombinant human CD36/SR-BIII Fc chimera, recombinant human galectin-3 protein, and human galectin-3 antibody (Monoclonal mouse IgG2B clone) were purchased from R&D systems (Minneapolis, MN, USA). HRP conjugated anti-mouse polyclonal goat (P0447) and polyclonal rabbit anti-mouse antibody/biotinylated were obtained from Dako (Glostrup, Denmark). Goat Anti-Human IgG/HRP was purchased from SouthernBiotech (Birmingham, MN, USA). TMB substrate (3,3′,5,5′-tetramethylbenzidine) for high sensitivity ELISA and streptavidin-PolyHRP80 were purchased from sdt-reagents (Baesweiler, Germany). Ovalbumin (OVA) was purchased from InvivoGen (San Diego, CA, USA). Bovine Serum Albumin Fraction V (BSA) was obtained from Roche (Basel, Switzerland). Amyloid-β (1–42) ultra-pure HFIP was purchased from Westburg (Leusden, The Netherlands). All other chemicals were purchased from Merck (Darmstadt, Germany).

### 4.2. CML Introduction in BLG

CML was introduced in BLG using the method by Glorieux et al. [25] with slight modifications. Briefly, BLG was dissolved in 10 mM phosphate saline buffer (PBS) at pH 7.4. Glyoxylic acid monohydrate was added in three different molar ratios of glyoxylic acid to lysine residues. These ratios were 1:1, 3:1, and 5:1, respectively. Subsequently, sodium cyanoborohydride was added in a ratio 8.8 mmol/g BLG and the solutions were incubated for 20 h at 40 °C in a heating block (Labtherm Graphit, Liebisch, Germany). Incubation of BLG at 40 °C for 20 h was performed as control (C1). After incubation, samples were dialysed at 4 °C using a MWCO of 6–8000 Spectra/Por membrane (, Thermo Fisher Scientific, Waltham, MA, USA) to remove residual glyoxylic acid. All samples were prepared in duplicate. All solutions were freeze dried and dissolved in water.

### 4.3. Glycation of β-lactoglobulin

BLG was glycated with lactose at 60 °C to minimise thermally induced protein aggregation. Incubation was conducted over saturated potassium fluoride solution to keep the water activity (a_w_) at 0.23. Heating was conducted for 12 h, 24 h, and 48 h in the presence of lactose. BLG heated under the same conditions in the absence of lactose was taken as heating control. After heating, BLG was immediately cooled down and dissolved in water before storage at −20 °C and stored until further analysis.

### 4.4. Quantification of CML Using LC-MS/MS

CML was quantified after borohydrid reduction and acidic hydrolysis and solid phase extraction were conducted considering previous methods [52,53]. Quantification was conducted using external calibration with d2-CML as internal standard. Analysis was performed using a Nexera UPLC system (Shimadzu Corporation, Kyoto, Japan) coupled with a LCMS-8050 triple quadrupole mass spectrometer (Shimadzu Corporation, Kyoto, Japan). The UPLC unit consisted of a SIL-30AC autosampler, a LC-20ADXR solvent delivery module, DGU-20ASR degassing unit, a CTO-20AC column oven and a FCV-20AH2 valve unit. The samples (5 µL) were injected on a Kinetex HILIC 2.6 µm, 2.1 mm × 100 mm (Phenomenex, Torrance, CA, USA). The flow rate was set at 0.4 mL/min and the column temperature at 30 °C. The mobile phases consisted of 0.1% formic acid (solvent A), acetonitrile with 0.1% formic acid (solvent B) and 50 mmol/L ammonium formate (solvent C) with the following elution profile (time in [minutes]/[%B]/[%C]): (0/80/10), (0.8/80/10), (3.5/40/10), (6.5/40/10), (8.0/80/10), and (10.0/80/10). MS data was collected for 10 min. Positive ionisation mode was used for all MS analyses. The voltage of the turbo ion-spray ionization was 4.0 kV. The temperature of electrospray ionization probe, desolvation line, and heat block were set at 300 °C, 250 °C, and 400 °C, respectively. The pressure of the collision-induced dissociation gas was 4 kPa whereas the flow rates of the drying gas, nebulizer gas, and heating gas were set at 10 mL/min, 3 mL/min, and 10 mL/min, respectively. The electrode voltage of Q1 pre bias (collision cell energy entrance potential), collision cell Q2 (collision energy), Q3 pre bias (collision cell energy exit potential), parent and fragment ion m/z of the multiple reaction monitoring transitions were optimized using support software (Shimadzu Corporation, Kyoto, Japan). Precursor ions were 205,200 m/z and 207,200 m/z for CML and CML-d_2_, respectively. Product ions were 84,050 m/z, 130,050 m/z, 56,150 m/z and 84,050 m/z, 130,200 m/z, 56,100 m/z for CML and CML-d_2_, respectively. The second and third product ion yield was selected as structural confirmation based on the optimized SRM transition, whereas the first product ion was used for quantification.

### 4.5. Quantification of Free Available Amino Groups

Free amino groups were measured using OPA-assay to monitor overall formation of MPRs other than CML. OPA-assay was conducted following a method published by Mulet-Cabero et al. [54] with some modifications. Samples were diluted with 10 mM PBS and 10 µL were pipetted in a transparent polystyrene 96-well plate (Greiner, Kremsmünster, Germany). To each well, 190 µL OPA reagent was added and the plate was incubated in the dark for 15 min. L-Leucine was used for the calibration curve in a concentration range between 0.4 to 4 mM. Absorbance was measured at 340 nm using Infinite^®^ 200 PRO NanoQuant with i-control software (Tecan, Männedorf, Switzerland).

### 4.6. Secondary Structure

Secondary structure was analysed with far UV-CD. Measurements were conducted using a Jasco J-715 spectropolarimeter (Jasco, Tokyo, Japan). Samples were diluted to 0.15 mg/mL protein concentration. The secondary structure was recorded with far UV between a range of 180 to 260 nm using a 15 times quartz cuvette with a path length of 0.1 cm at 20 °C.

### 4.7. Surface Hydrophobicity (ANS-Assay)

Surface hydrophobicity was determined using ANS–assay according to a method Alizadeh-Pasdar et al. [55] with some modifications. Samples were diluted to 0.5 mg/mL and mixed with 0.8 mM ANS solution in a ratio 1/20 (*v/v*). After incubation for 10 min in the dark, fluorescence was measured with an excitation wavelength of 390 nm and emission wavelength of 470 nm.

### 4.8. ThT-Assay

The ThT-assay was performed to determine a possible increase in amyloid-β structures. Samples were diluted to a protein concentration of 2 mg/mL with 10 mM PBS (pH 7.4). Subsequently 20 µL sample were mixed with 20 µL aqueous ThT-solution (1.25 mg/mL ThT) and mixed with 120 µL PBS. Samples were incubated for 10 min at room temperature in the dark. Fluorescence intensity was measured with an excitation wavelength of 450 nm and emission wavelength of 480 nm. All samples were measured in duplicate and corrected for the blank (PBS with ThT-solution). To correct for autofluorescence samples were measured at the same dilution as in the assay and signal was subtracted when applicable.

### 4.9. Native Gel Electrophoresis

Native gel electrophoresis was performed to monitor possible formation of aggregates in the samples. Samples were diluted to equal protein concentrations in 10 mM PBS (pH 7.4) and mixed with native samples buffer (1:1 (*v/v*)). Those solutions were loaded onto the gel with a final protein content of 10 µg in each pocket. The gels were run at 120 V for 90 min in 1x native running buffer and subsequently stained with Coomassie brilliant blue R-250. Images were taken using a Universal Hood III (Biorad, Hercules, CA, USA) and Image Lab 4.1 software (Biorad, Hercules, CA, USA).

### 4.10. Inhibition ELISA Assay for Receptor Binding

Binding to the receptors for AGEs was determined with inhibition ELISA as described by Zenker et al. [29]. Briefly, samples were diluted with 10 mM PBS to 100 µg/mL protein concentration. Free CML was measured at a concentration of 2 mg/mL. Dilutions were incubated for 45 min at 37 °C on a Nunc™ 96-Well Polypropylene MicroWell™ (Thermo Fisher Scientific, Waltham, MA, USA) with the receptors at concentrations of 0.5 µg/mL for sRAGE, 0125 µg/mL for CD36, and 0.4 µg/mL for galectin-3, respectively. Subsequently, the solutions were added to a high binding transparent Nunc-Immuno™ MicroWell™ 96-well solid plate (Thermo Fisher Scientific, Waltham, MA, USA), blocked with soy protein glycated with glucose for 90 min at 100 °C (G90) and incubated for 1 h at 37 °C. After washing, the plates were incubated for 30 min at room temperature with the specific anti-receptor antibody: anti-RAGE, anti-galectin-3, or anti-his antibody for CD36. Subsequently incubation for 30 min at room temperature was performed with the respective detection antibody: polyclonal goat anti-mouse Antibody/HRP, polyclonal rabbit anti-mouse antibody/ biotinylated, and goat anti-human IgG/HRP. For galectin-3, an additional incubation step was performed with streptavidin for 15 min at room temperature before detection with 3,3′,5,5′-tetramethylbenzidine solution. Absorbance was measured at λ = 450 nm with λ = 620 as reference wavelength. To evaluate the role of charge in binding to the receptors for AGEs different concentrations of NaCl were used. The dilution buffer for the samples contained 0.13 M NaCl. This was supplemented to reach 0.2 M and 0.3 M NaCl in the final solution of the receptor/sample mixture, respectively. All samples were measured in technical triplicates.

## 5. Conclusions

Chemical modification of BLG to introduce CML on lysine residues resulted in a CML concentration-dependent increase of binding to sRAGE, CD36, and galectin-3, confirming that protein-bound CML is a ligand for these AGE receptors. Next to BLG-CML, BLG-Lac-48 was also a potent ligand for the AGE receptors, which was positively correlated with increasing hydrophobicity upon longer heating time of BLG-Lac. The reduced receptor binding upon increased salt concentration indicated that the negative charge induced to the lysine residues by CML was a crucial determinant for binding of BLG-CML samples to AGE receptors. The same effect of salt concentration was also observed for the BLG-Lac-48 sample suggesting that, next to hydrophobicity other MRPs that induce a negative charge to lysine residues can contribute to the binding of glycated proteins. These findings showed that heating and glycation of food proteins results in structural modifications amongst which hydrophobicity and MRPs carrying a negative charge were found to be important determinants to predict possible immunological consequences. This also highlights the possible physiological relevance of CML in processed food as well as other MRPs that induce a negative charge to amino acid residues. At the same time, bioavailability of these MRPs and the relevance in aetiology of adverse inflammation in vivo remains to be better investigated.

## Figures and Tables

**Table 1:** CML content of differentially treated β-lactoglobulin (BLG). BLG was non-treated (NT), chemically modified with glyoxal to obtain Nε-carboxymethyl lysine (CML) at different degree of modification (1, 3, and 5), glycated with lactose (Lac) by heating at 60 °C for 12, 24, and 48 h; heated at 60 °C for 12, 24, and 48 h in the absence of any sugars (H), and glycated by heating with lactose followed by CML induction with glyoxal. CML modified lysine was calculated based on theoretical levels of lysine available in BLG. Results are given as mean ± standard deviation (*n* = 2). Statistical differences were tested using one-way ANOVA with Tukey post-hoc test. Significant differences are considered at *p* < 0.05, where two variables have different letters if they are significantly different.

Sample	CML Content [mg/g Protein]	CML Modified Lysine [%]
BLG-NT	0.4 ± 0.3 a	0.39 ± 0.3 a
BLG-CML-1	28.0 ± 0.1 b	17 ± 0.0 b
BLG-CML-3	56.1 ± 0.1 cd	34 ± 0.1 de
BLG-CML-5	62.6 ± 1.3 d	38 ± 0.8 d
BLG-Lac-12	2.0 ± 0.2 a	1.2 ± 0.1 a
BLG-Lac-24	3.7 ± 0.4 a	2.2 ± 0.2a
BLG-Lac-48	4.4 ± 0.3 a	2.7 ± 0.2 a
BLG-H-12	1.6 ± 1.9 a	1.0 ± 1.2 a
BLG-H-24	0.3 ± 0.0 a	0.2 ± 0.0 a
BLG-H-48	0.4 ± 0.2 a	0.2 ± 0.1 a
BLG-Lac-12-CML	46.4 ± 2.9 c	28 ± 1.8 c
BLG-Lac-24-CML	50.3 ± 7.8 c	31 ± 4.7 cd
BLG-Lac-48-CML	34.5 ± 1.9 b	16 ± 0.9 b

## Supplementary Materials

Supplementary materials can be found at https://www.mdpi.com/1422-0067/21/12/4567/s1.

## Abbreviations

AGEs	Advanced glycation end products
ANS	8-anilino-1-naphthalenesulfonic acid
APCs	Antigen presenting cells
BLG	β-lactoglobulin
BSA	Bovine serum albumin
CD	Circular dichroism
CML	Nε-carboxymethyl lysine
MR	Maillard reaction
MRPs	Maillard reaction products
NaCl	Sodium chloride
OPA	o-phthaldialdehyde
oxLDL	Oxidised low density lipoprotein
PBS	Phosphate saline buffer
RAGE	Receptor for advanced glycation end products
sRAGE	Soluble form of RAGE
ThT	Thioflavin T
