# Supplementary material for: Binding of CML-Modified as Well as Heat-Glycated β-lactoglobulin to Receptors for AGEs Is Determined by Charge and Hydrophobicity
Source: Int J Mol Sci. 2020 Jun 26;21(12):4567. doi: 10.3390/ijms21124567 (PMC7348724; doi:10.3390/ijms21124567)
Supplement: Supplementary file 1 [file ijms-21-04567-s001.pdf]

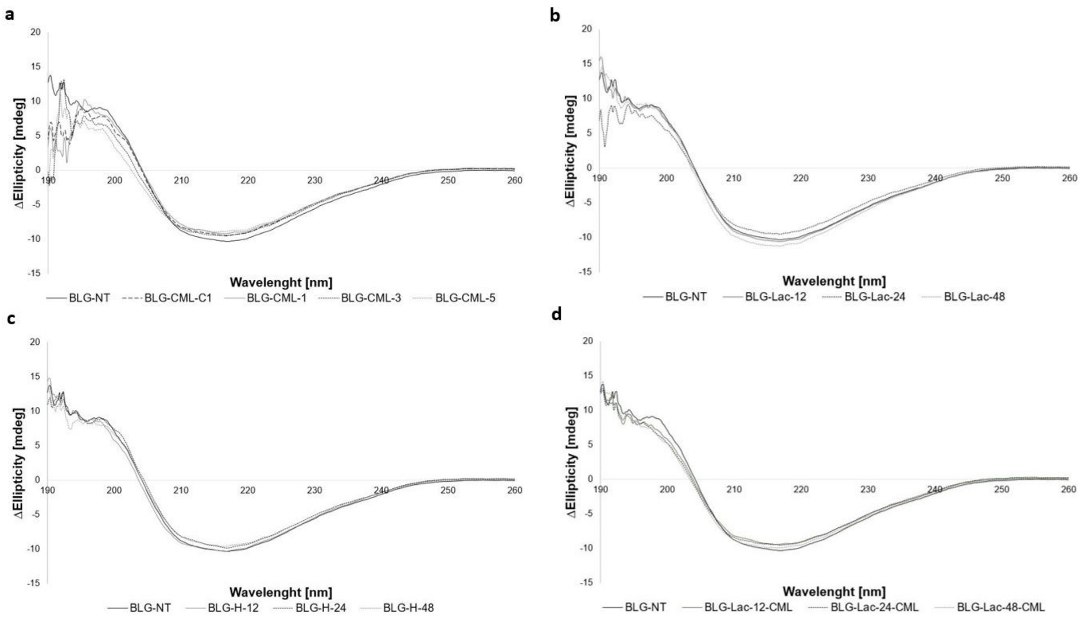

Figure S1. Far-UV circular dichroism spectra of (a) β-lactoglobulin (BLG), non-treated (NT), chemical modification control without addition of glyoxylic acid (C1), chemically modified to introduce Nε-carboxymethyl lysine (CML) at different degree of modification (1, 3, and 5), (b) BLG glycated with lactose (Lac) at 60 °C for 12, 24, and 48 hours, (c) BLG heated in the absence of lactose (H) at 60 °C for 12, 24, and 48 hours, and (d) BLG glycated and subsequently chemically modified.
